# Construction and validation of a matrix for normative evaluation of the integrated health system of the borders*

**DOI:** 10.1590/1518-8345.4141.3433

**Published:** 2021-07-02

**Authors:** Luciana Aparecida Fabriz, Valéria Conceição de Oliveira, Fabiana Costa Machado Zacharias, Silvia Helena Valente, Denise Ferro, Ione Carvalho Pinto

**Affiliations:** 1 Universidade Estadual do Oeste do Paraná, Campus Foz do Iguaçu, Foz do Iguaçu, PR, Brazil.; 2 Universidade São João Del Rei, Campus Divinópolis, Divinópolis, MG, Brazil.; 3 Universidade de São Paulo, Escola de Enfermagem de Ribeirão Preto, PAHO/WHO Collaborating Centre for Nursing Research Development, Ribeirão Preto, SP, Brazil.

**Keywords:** Validation Studies, Program Evaluation, Public Health Policy, Health Systems, Border Areas, Border Health, Estudos de Validação, Avaliação de Programas e Projetos de Saúde, Políticas Públicas de Saúde, Sistemas de Saúde, Áreas de Fronteira, Saúde na Fronteira, Estudios de Validación, Evaluación de Programas y Proyectos de Salud, Políticas Públicas de Salud, Sistemas de Salud, Áreas Fronterizas, Salud Fronteriza

## Abstract

**Objective:**

to build and validate a matrix for normative evaluation of the Integrated Health System of Borders.

**Method:**

a methodological study, composed by the construction of an evaluation matrix elaborated in three stages: elaboration of the logical model, containing the triad of structure, process and result; definition of evaluative questions and appearance and content validation of the matrix. Appearance and content validation were performed simultaneously by seven judges. For data collection, an online questionnaire and the Delphi technique were used and, for analysis, the Content Validity Index and Content Validity Ratio.

**Results:**

the evaluation matrix containing 24 questions was submitted to two evaluations for its appearance and content validation. In the first, the overall mean Content Validity Index was 99.40% and the Content Validity Ratio was 0.90. In the second, the Content Validity Index was 100% and the Content Validity Ratio, 1.0; there were no new proposals and the matrix was made up of 24 questions. The matrix was considered intelligible in terms of appearance validation.

**Conclusion:**

the evaluation matrix of the Integrated Health System of the Borders is validated in terms of appearance and content for analyzing the performance of public actions and policies in border regions.

## Abstract (pt)

**Objetivo:**

construir e validar uma matriz para avaliação normativa do Sistema Integrado de Saúde das Fronteiras.

**Método:**

estudo metodológico, composto pela construção de uma matriz avaliativa elaborado em três etapas: elaboração do modelo lógico, contendo a tríade estrutura, processo e resultado; definição de questões avaliativas e validação aparente e de conteúdo da matriz. A validação aparente e de conteúdo foi realizada simultaneamente por sete juízes. Para a coleta de dados utilizou-se questionário *on-line* e a técnica Delphi, e para análise, o Índice de Validade de Conteúdo e Razão de Validade de Conteúdo.

**Resultados:**

a matriz de avaliação contendo 24 questões foi submetida a duas avaliações para a sua validação aparente e de conteúdo. Na primeira, o Índice de Validade de Conteúdo médio geral foi de 99,40% e a Razão de Validade de Conteúdo de 0,90. Na segunda, o Índice de Validade de Conteúdo foi de 100% e a Razão de Validade de Conteúdo de 1.0, não houve novas proposições e a matriz foi constituída de 24 questões. A matriz foi considerada inteligível quanto à validação de aparência.

**Conclusão:**

a matriz avaliativa do Sistema Integrado de Saúde das Fronteiras encontra-se validada quanto à aparência e conteúdo para análise do desempenho de ações e políticas públicas em regiões de fronteira.

## Abstract (es)

**Objetivo:**

construir y validar una matriz para evaluar la normativa del Sistema Integrado de Salud de las Fronteras.

**Método:**

estudio metodológico, compuesto por la construcción de una matriz de evaluación elaborado en tres etapas: elaboración del modelo lógico, que contiene tres componentes: estructura, proceso y resultado, definición de cuestiones evaluativas y validación de aspecto y de contenido de la matriz. Siete jueces realizaron la validación de aspecto y de contenido en forma simultánea. Para la recolección de datos se utilizó un cuestionario online y la técnica Delphi y, para el análisis, el Índice de Validez de Contenido y la Relación de Validez de Contenido.

**Resultados:**

se sometió la matriz de evaluación con 24 preguntas a dos evaluaciones para su validación de aspecto y de contenido. En la primera, el Índice de Validez de Contenido medio general fue del 99,40% y la Relación de Validez de Contenido fue de 0,90. En la segunda, el Índice de Validez de Contenido fue del 100% y la Relación de Validez de Contenido fue de 1,0; no hubo nuevas propuestas y la matriz quedó constituida por 24 preguntas. Se consideró la matriz inteligible respecto a la validación de aspecto.

**Conclusión:**

la matriz de evaluación del Sistema Integrado de Salud de las Fronteras se encuentra validada en cuanto a aspecto y contenido para el análisis del desempeño de acciones y políticas públicas en regiones de frontera.

## Introduction

The concept of borders has been improved over the years, ceasing to be seen only as barriers, and becoming contact zones and places for regional integration^(^ 1 ^)^.

In the international borders, between the local and the international space, it is where the border peoples are located, which are the people who live, articulate and establish bonds. In this context, pendular migration takes place, which consists of people moving from their country of origin to the neighboring country, for work, study, or other reasons^(^ 2 ^)^.

The mobility of people across international borders is a worldwide phenomenon and can cause divergences when the countries present economic discrepancies, as is the case of the United States of America (USA), which adopts rigid standards on the border with Mexico, due to the Mexicans searching for better living conditions in the USA, in addition to the flow of illegal drugs and due to trade; in contrast, this problem does not occur on the border between the USA and Canada, as the countries have economic equivalence^(^ 3 ^)^.

In the case of Brazil, the fact that it has a free national health system has encouraged the migration of people from other Latin American countries to seek medical care; this phenomenon has caused difficulties in controlling and eliminating diseases such as leprosy^(^ 4 ^)^. The surplus foreign population is not counted for financial transfer in border municipalities^(^ 5 ^)^.

Public policies for borders and transnational integration are recent; previously, they were restricted to security and occupation of the territory. Only in 2005 did the Ministry of Health (MoH) launch the Integrated Border System (SIS Fronteiras) in order to carry out a local health diagnosis, promote the qualification of the professionals, and improve the health service network in border municipalities, effective from 2005 to 2014^(^ 6 ^)^. However, the execution of SIS Fronteiras did not end in 2014; the activities extended over the following years, as planned by each municipality to meet the established goals. 

It is considered that the evaluation of public actions and policies in border regions is important for facing the local problems, as well as for regional development^(^ 6 ^)^. This study aimed to build and validate a matrix for normative evaluation of *SIS Fronteiras*.

## Method

A methodological study, in a border region, elaborated in three stages: elaboration of the logical model, containing the triad of structure, process and result; definition of evaluation questions and appearance and content validation of the matrix.

To identify the knowledge gaps and survey the scientific research studies and legislation relevant to *SIS Fronteiras*, a Scoping Review literature was carried out, in five stages: identification of the research question; identification of relevant studies; study selection; data mapping and grouping; summary and presentation of results^(^ 7 ^)^. Research studies and documents available until 2017 were included, in the Medical Literature Analysis and Retrieval System on-line (MEDLINE/PubMed), Latin American and Caribbean Literature in Health Sciences (*Literatura Latino-Americana em Ciências de Saúde*, LILACS), SciELO (Scientific Electronic Library Online), EMBASE, Web of Science, Cummulative Index to Nursing and Allied Health Literature (CINAHL); Journals Portal of the Coordination for the Improvement of Higher Level Personnel (*Coordenação de Aperfeiçoamento de Pessoal de Nível Superior*, CAPES); Elsevier's Scopus Database; Health Legislation System of the Brazilian Ministry of Health; Virtual Health Library of the Brazilian Ministry of Health; Brazilian Ministry of Foreign Affairs, and Google Scholar.

The following search strategies were used: ("Sistema Integrado de Saúde das Fronteiras" OR "Sistema Integrado de Saúde da Fronteira" OR "Sistema Integrado de Saúde nas Fronteiras" OR "Sistema Integrado de Saúde na Fronteira" OR "SIS-Fronteiras" OR "SIS-Fronteira") OR ("SIS Border" OR "SIS Frontiers" OR "SIS-Borders Project"). The results found in the Scoping Review demonstrated the non-existence of a validated matrix for the evaluation of *SIS Fronteiras* and made it possible to identify elements such as human resources, financing, cooperation terms, and local health diagnoses, among others. The data were organized in structure, process and results and supported the development of the logical model, which guided the construction of the evaluation matrix for *SIS Fronteiras* ^(^ 8 ^)^.

Based on the logical model, evaluation questions were identified, which were later validated by judges, according to the criteria of clarity, objectivity and relevance^(^ 9 ^)^. The validation of the content and appearance of the questions took place from November 2017 to February 2018, using the Delphi Technique, described below^(^ 10 ^-^ 11 ^)^.

The study population was composed of seven judges with expertise on the subject matter, who simultaneously participated in content and appearance validation and were selected through an advanced search in the Lattes Platform curriculum, a database of the National Council for Scientific Development (*Conselho Nacional de Desenvolvimento Científico*, CNPq). For the survey on the platform, the search mode chosen was by subject matter and the following keywords were used: *SIS Fronteiras* (SIS Borders), *Saúde nas Fronteiras* (Health at the Borders) and *Fronteira* (Border), with the boolean logical operator "AND" between the words. The contacted researchers were able to appoint other researchers to replace them if necessary or to increase the number of participants. 

The inclusion criteria were the following: being a teacher, researcher or professional with knowledge regarding *SIS Fronteiras*; having a college degree, and having an active e-mail account. Participants with automatic e-mail responses, informing inability to respond due to vacation, leave or other reasons and those who sent out questionnaires after 15 days from receipt were excluded.

Prior contact for the selection of judges was established via e-mail, in order to invite them to participate in the research. In this contact, the objective, method, and justification of the study were presented and it was explained that participation would occur individually and remotely, through an on-line questionnaire, electronic form (Google Forms application), simultaneously contemplating content and appearance validation. After the acceptance of each judge, an e-mail containing the Free and Informed Consent Form (FICF) was sent, which was signed and returned by each participant, as well as the link to access the online questionnaire. 

The questionnaire was designed with 24 evaluative questions, eight of which were related to structure, eight to process and eight to result, which were subsequently subjected to content and appearance validation by judges.

For the evaluation of the questions, a five-point Likert scale was used, being 1 (totally disagree), 2 (partially disagree), 3 (neither disagree nor agree), 4 (partially agree) and 5 (totally agree). The judges were given the option to suggest alternative texts on each question. The deadline for returning the evaluation matrix was 15 days from receipt^(^ 10 ^)^.

A 75% agreement percentage was considered for each evaluated item; in the case of failure to obtain the degree of agreement between the judges, new cycles should be carried out^(^ 11 ^)^. At the end, a thank you e-mail was sent to the participants for their collaboration in the research.

Content validity was carried out to analyze the completeness of the proposed items and whether they reflected the theoretical framework and the *SIS Fronteiras* guidelines, in addition to appearance validation because, despite not being a sophisticated technique, it was intended to observe how content was presented, as well as clarity and ease of reading and the adequacy of the item to each dimension^(^ 9 ^,^ 12 ^)^.

For data analysis, the Content Validity Index (CVI) was used to measure the degree of agreement between the judges based on their answers. The expected consensus level for this study was 75%^(^ 11 ^)^.

The CVI was calculated from the sum of answers 4 and 5 (partially agree and totally agree) of each judge in each question of the questionnaire, dividing this sum by the total number of answers (CVI=Number of answers 4 and 5/Number total of responses X 100). The mean CVI of the dimensions was also calculated, adding each percentage obtained in each dimension and dividing by three, that is, by the three dimensions used.

Content Validity Ratio (CVR)^(^ 13 ^)^ was also used. The CVI measures the proportion of judges with answers 4 and 5 (partially agree and strongly agree) and the CVR compares this proportion with the expected number if the judges were responding by chance [CVR=ne-(N/2)/(N/2)], where "ne" is the number of judges who rated each item as 4 and 5, while "N" is the total number of respondent judges.

The CVI varies between 0 and 1; the closer to 1, the better the item will perform according to the judges. The CVR varies between -1 and 1, the minimum value depending on the number of judges. In this study it was considered as 0.99^(^ 13 ^)^.

Appearance validation was performed considering the requirement for clarity of content and the semantics of the questions; thus, it was verified that all items were comprehensible to the respondent population, with opportunities for descriptive manifestations for each question^(^ 14 ^)^. 

The project was approved by the Committee of Ethics in Research with Human Beings, CAAE: 68748617.3.0000.5393.

## Results

**Figure 1:**
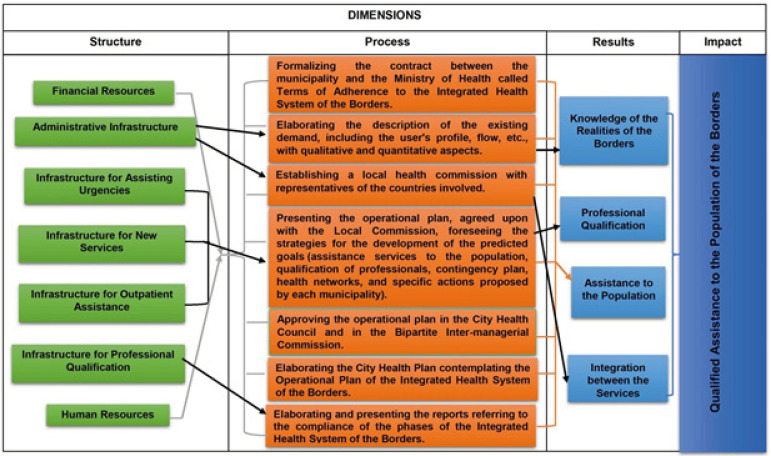
Logical Model of the Integrated Health System of the Borders. Ribeirão Preto, SP, Brazil, 2018

In the logical model, the necessary elements for the development of *SIS Fronteiras* were considered, such as: structural - financial resources (amounts transferred); administrative infrastructure (room for coordination); infrastructure for emergency care, outpatient care and new services (room, equipment and materials); infrastructure for professional qualification (computers, bibliographic material) and human resources (staff assigned and/or hired), process - the operational actions for the approval and maintenance of the program, such as: formalization of the Term of Adherence to *SIS Fronteiras*; description of the clientele considering assistance flows and user profile; local health commission; operational plan; municipal health plan and reports. The following were considered as results: knowledge of the border realities; qualified professionals; service provided to the population and integration between the services. After the elaboration of the evaluative design, based on the scientific literature and the regulations, the expected impact was defined, as the qualified service to the border population (Figure 1). 

Based on the logical model (Figure 1), 24 evaluative questions were initially identified, eight of which were related to structure, eight to process and eight to result, which were subsequently submitted to content and appearance validation by judges.

In the validation stage, 18 researchers were identified in a previous selection by the keywords. After applying the other inclusion and exclusion criteria, ten were selected, of which three contributed with the pilot test of the evaluation matrix and seven judges effectively participated in the validation process: five female and two male. As for the training area: three were from Nursing, one from Biomedicine, one from Dentistry, one from Psychology and one from Law; of these, two were PhDs and five were MSs.

The evaluation matrix was subjected to two rounds in the validation process and, in the first round, in the structure and process dimensions, the mean CVI was 100% and the CVR was 1.0, while in the result dimension, the mean CVI was 98.21% and the CVR was 0.96. Despite reaching a consensus in the first round, it was sent to the second round for the purpose of presenting a new version, containing the suggestions of the judges and justification for the non-changes. In the second round, there were no new proposals and the CVI for each dimension was 100%, with a CVR of 1.0.

The overall mean CVI of the evaluation matrix, considering the three dimensions (structure, process and result), was 99.40% and the CVR was 0.90. In the second round, there were no new proposals; therefore, 100% agreement and a CVR of 1.0 were obtained.

With regard to appearance validation, the judges presented two proposals for the structure dimension; two suggestions for process; and a recommendation for the result dimension. The suggestions were analyzed and accepted with the necessary adjustments, as shown in Figure 2.

**Figure 2:** Statements of the suggestions presented in the appearance validation and the respective changes or justifications for the non-changes. Ribeirão Preto, SP, Brazil, 2018

Rounds	Suggestions and changes or justifications
	Structure
1^st^	Here, in question 5, which refers to "room, equipment and materials for the first assistance of urgencies for 100% of the described population, contemplating", it was necessary to say where it is described and what should be contemplated.
2^nd^	The question was revised and the information referring to the documents that should include the description of the population was included and the word "contemplating" was excluded, since it is already stated that it was equipment and materials.
1^st^	In question 5, there is the word *urgencies*, I think it is not necessary, as it says that it is for first care.
2^nd^	In this case, the word *urgencies* was maintained, for clarification to the reader, as the agreed service was for the first assistance of urgency cases.
	Process
1^st^	In question 10, regarding the municipality to elaborate the client description, within 30 days from the Term of Adherence, I suggest extending the term to 30 to 40 days.
2^nd^	The item was not changed, considering that the term of up to 30 days was established under Ordinance 1,189 of June 5th, 2006.
	Process
1^st^	In question 15, which deals with the insertion of *SIS Fronteiras* in the Municipal Health Plan, it is necessary to clarify which period it refers to as, since the implementation of SIS Fronteiras in 2006, there are already at least four plans prepared in that period, with this, I suggest that they are in the Municipal Health Plans.
2^nd^	Suggestion accepted, changing the text to: "The Municipal Health Plans, elaborated during the term and execution of *SIS Fronteiras*, contain the Local Diagnosis and the Operational Plan".
	Results
1^st^	In question 23, there is a contingency plan for unusual events, but they were not exclusive to *SIS Fronteiras*, I think they could be removed.
2^nd^	The contingency plan for unusual events is a requirement for the release of financial resources from *SIS Fronteiras*; therefore, it was maintained.

After the necessary adjustments and conclusion of the validation process, the matrix for normative evaluation of *SIS Fronteiras* was constituted of 24 evaluation questions, as shown in Figure 3 below.

**Figure 3:** Statement of the questions regarding the evaluation matrix of SIS Fronteiras. Ribeirão Preto, SP, Brazil, 2018

Structure
1. The Ministry of Health allocated financial resources for the development of SIS Fronteiras.
2. The municipality provided a room for the coordination of the project, containing 1 computer; 1 printer; 1 nobreak; 1 table; 1 typing chair and 1 cabinet.
3. The municipality provided infrastructure for the implementation of health networks, including: computers; information systems; primary and tertiary care points, to support primary health care, with specialized actions at outpatient and hospital levels, diagnostic and therapeutic support, meeting room and car for commuting.
4. The municipality provided offices, with equipment, furniture and materials for basic outpatient care.
5. The municipality provided a room, equipment and materials for first emergency care to 100% of the population described in the *SIS Fronteiras* Term of Adherence and Operational Plan.
6. Infrastructure, made available by the municipality, for new services proposed in the Local Health Diagnosis, which may include constructions; reforms; acquisition of equipment; vehicle purchase; consumables, etc.
7. Infrastructure, provided by the municipality, for qualification, contemplating the following: Physical space (available rooms for qualification), Computers and Bibliographic material.
8. Personnel, assigned and/or hired by the municipality for the activities inherent to *SIS Fronteiras*, with the possibility of hiring professionals and paying charges when provided for in the Operational Plan.
Process
9. The Municipality formalized a partnership with the Ministry of Health, through a contract called Term of Adherence to the Integrated Health System of the Borders, *SIS Fronteiras*, within the established term (April 2006).
10. The municipality prepared and presented the clientele description, within up to 30 days from the manager's signature on the Term of Adherence to *SIS Fronteiras*.
11. The municipality constituted a Local Health Commission in an integrated manner with the Municipal Health Council and with the participation of representatives of the health systems on both sides of the border, within 30 days of the manager's signature on the Term of Adherence to *SIS Fronteiras*.
12. The municipality presented an Operational Plan within 60 months from the manager's signature on the Term of Adherence to *SIS Fronteiras*.
13. The Municipal Health Council approved the Local Health Diagnosis and the Operational Plan.
14. The State Bipartite Inter-Management Commission approved the Local Health Diagnosis and the Operational Plan.
15. The Municipal Health Plans, elaborated during the validity and execution of SIS Fronteiras, include the Local Health Diagnosis and the Operational Plan.
16. Reports on the Phases of *SIS Fronteiras* were forwarded.
Results
17. Local Health Diagnosis complete, with qualitative and quantitative aspects, contemplating the identification of the population to be served in the health services; survey of existing demands; installed capacity; description of assistance flows; definition of the epidemiological, sanitary and environmental profile in health.
18. Qualified professionals in terms of management (economics, planning, organization of the health systems and supplementary health management), health care (protocols and specific border procedures), health surveillance, indigenous health, information systems and social control.
19. Health networks implemented, respecting the financial limits.
20. New services implemented according to demands surveyed in the Local Health Diagnosis.
21. Basic outpatient care available to 100% of the population described in the Local Health Diagnosis and Operational Plan.
22. Urgent care available to 100% of the population described in the Local Health Diagnosis and Operational Plan.
23. The municipality presented the Contingency Plan for Unusual Events.
24. The actions listed by the manager in the Local Health Diagnosis and Operational Plan were carried out.

For the evaluation matrix, a Likert-type scale was also prepared and validated, adapted to each question, where 0 corresponds to non-compliance with the guidelines (the manager did not perform any action and/or did not present any documents); 1 to partially met guidelines (manager performed only a few actions and/or presented part of the documents); 2 to guidelines inappropriately met (the manager carried out the actions and presented all the documents, but part of them were inadequately net); and 3 to full compliance with the *SIS Fronteiras* guidelines (the manager developed all the actions and delivered all the required documents).

## Discussion

The analysis of scientific documents and articles through the Scoping Review provided consistent information for the elaboration of the logical model, which supported the construction of the questions that made up the evaluative matrix of *SIS Fronteiras*. The construction of the logical model is a very important stage in the evaluation of programs and/or public policies, as it constitutes the representation of its functioning and favors the understanding of its projects and actions; its applicability has been used in the evaluation of important programs such as More Doctors and Leprosy Control^(^ 15 ^-^ 16 ^)^.

The matrix constructed and validated in this study obtained a positive evaluation, with an overall mean CVI of 100% for the three dimensions, namely: structure, process and results; and CVR of 1.0, therefore being considered adequate and superior to the established 75% cutoff. In the literature, the cutoff point for reaching consensus among judges ranges from 50% to 80%, for Nursing research, a percentile greater than 75%^(^ 11 ^)^ has been considered.

In the appearance validation, the matrix was considered intelligible, after the adjustments suggested by the judges. The main recommendations presented were related to the clarity of the content and the semantics of the questions. A number of studies point out the importance of semantic evaluation because, at this moment, the judges make sure that the items are written in a clear and understandable way for the target population, avoiding doubts and ensuring appearance validity^(^ 14 ^,^ 17 ^)^. 

In validated matrix aspects of structure, process and result were addressed and it will allow managers, researchers and health professionals to have knowledge on the process of implementing *SIS Fronteiras* in each location, enabling the identification of the potentialities and weaknesses of this public health policy.

In the structure dimension, in the first round, the CVI was 99.40% and the CVR was 0.90 and, in the second round, there were no new proposals, so 100% agreement and a CVR of 1.0 were obtained. In this dimension, important points were considered for the development of *SIS Fronteiras*, such as infrastructure, financial, human and material resources. The structural elements included in the evaluation matrix converge with national studies that point out that insufficient financial resources, deficit of employees, and the precarious physical structure are problems faced by the managers of border municipalities. This is due to the asymmetry of the health systems of the countries bordering Brazil and to the demand of foreigners for health care; and *SIS Fronteiras*, through financial transfer, was a crucial strategy to improve the situation in these locations^(^ 18 ^)^. 

The Brazilian reality is similar to border regions of other countries in the world, as is the case of San Diego - USA and Tijuana - Mexico, where there are also differences with regard to aspects such as language, culture and economy, and thousands of people cross over to the other side daily, especially Mexican workers to work. In recent years, in San Diego - Tijuana, public and private non-profit organizations have promoted infrastructure and economic projects to expand the economic, social and political border relations^(^ 19 ^)^.

In the process dimension, in the first round a CVI of 99.40% and a CVR of 0.90 were obtained and, in the second round, there were no new proposals, resulting in 100% agreement and in a CVR of 1.0. In the proposals, the judges validated that the evaluative matrix should generally consider aspects related to the operationalization of the actions developed or planned and the integration between the countries neighboring Brazil, foreseen in *SIS Fronteiras*. 

The elements listed by the judges for the process dimension of SIS Fronteiras are similar to a study on cross-border cooperation at EU-Russia borders, in which the cross-border program was analyzed considering government coordination at various levels, with the purpose of implementing policies with mutual benefits; partnerships, creation of joint program management bodies; development of uniform policies and joint sources of project funding^(^ 20 ^)^.

For the result dimension, the mean CVI was 98.21% and the CVR was 0.96 and, in the second round, a CVI of 100% and a CVR of 1.0 were obtained, and the items validated by the judges refer to the identification of the achievement of the goals expected and the services implemented. The main goal foreseen in SIS Fronteiras is the elaboration of the Local Health Diagnosis, because it allows for a detailed analysis of the assisted population in border regions, especially the demand, related to the mobility of patients coming from neighboring countries seeking health care.

The mobility of patients to other countries for the purpose of health treatment, although it has always existed, has considerably increased in the last decade due to the greater commercial integration between different countries of the world. However, the main causes for patients crossing borders seeking health care are the difficulty of access or the lack of necessary services in their countries of origin^(^ 21 ^)^.

In addition to cross-border patients, who travel seeking health care and return to their countries, in this century, Brazil has experienced a considerable increase in the number of immigrants from several countries. This immigration is mainly motivated by economic, political and environmental issues. Recently, the case of the Venezuelans was evidenced, who are crossing the borders of northern Brazil as a way to escape the serious economic and political crisis in Venezuela^(^ 22 ^)^.

A study carried out in Costa Rica identified that the country also experiences a high demand from foreigners and people in cross-border transit and, in view of this situation, adopted the strategy to establish a shelter home for the purpose of humanitarian aid and the identification of this immigrant population^(^ 23 ^)^. 

In this light, in Brazil the local health diagnosis of SIS Fronteiras was pointed out by the judges, in this study, as an important evaluative requirement of this public policy and represented a relevant action for the identification of the foreign population. 

These aspects corroborate with a national study that considers that, in Brazil, there is no availability of accurate data regarding care in border regions, and that this information is often camouflaged and/or omitted by the patients to gain access to the services of the Unified Health System^(^ 24 ^)^.

The Delphi technique was presented as an important strategy for obtaining consensus among the judges and to conduct the necessary adjustments regarding the content and appearance of some questions in the evaluation matrix. The satisfaction and recognition of the relevance of the Delphi Technique for this study corroborates with other research studies developed with the use of this method, carried out through consensus of experts^(^ 25 ^)^.

Another potentiality of the Delphi technique in this study was the use of Google Forms, currently an online technology that is gaining prominence in scientific research, due to the ease of contacting people without having to travel and at lower costs. In this sense, research studies developed on the Delphi technique show that one of its advantages is the possibility of not being face-to-face, as it adds wealth to the study with the participation of specialists in specific themes from different geographical locations^(^ 26 ^-^ 27 ^)^.

Despite the numerous advantages perceived in the use of the method, we identified some limitations, such as the delay in acceptance and the difficulty in explaining the relevance of the study, without physical contact with the participant. Another limitation observed in the present study refers to the fact that Brazil has a vast territorial extension, with specificities in different regions; therefore, there may be certain difficulty in the applicability of the matrix throughout the national territory. 

Methodological studies are viewed with great relevance by the scientific community. Thus, this study, through the logical model and the evaluation matrix, has as strength the availability of a tool that will allow for the evaluation of *SIS Fronteiras* and, in this way, it can contribute to the formulation of new public health policies aimed at international standards of border regions.

## Conclusion

The normative evaluation matrix of SIS Fronteiras, elaborated and validated in this study, proved to be an innovative tool in the evaluation of public health policies in international border regions. 

The content and appearance validity of the matrix was considered adequate, in view of the careful process of analyzing its items and the suggestions to improve it, thus ensuring that the evaluation questions and the structure meet the *SIS Fronteiras* guidelines and are in accordance with the theoretical assessment approach.

The need to expand research studies related to health issues in border regions is highlighted, as well as the development of new tools to assess these very specific realities.
